# Hidden diversity of Nycteribiidae (Diptera) bat flies from the Malagasy region and insights on host-parasite interactions

**DOI:** 10.1186/s13071-017-2582-x

**Published:** 2017-12-29

**Authors:** Beza Ramasindrazana, Steven M. Goodman, Yann Gomard, Carl W. Dick, Pablo Tortosa

**Affiliations:** 1 Centre de Recherche et de Veille sur les maladies émergentes dans l’Océan Indien, Plateforme technologique CYROI, Sainte Clotilde, La Réunion, France; 2 Université de La Réunion, CNRS 9192, INSERM U1187, IRD 249, Unité Mixte Processus Infectieux en Milieu Insulaire Tropical (PIMIT), Plateforme Technologique CYROI, Sainte-Clotilde, La Réunion, France; 3 Association Vahatra, 101 Antananarivo, Madagascar; 4 Institut Pasteur de Madagascar, Ambatofotsikely, 101 Antananarivo, Madagascar; 5 Field Museum of Natural History, Chicago, IL 60605 USA; 6 Department of Biology, Western Kentucky University, Bowling Green, KY 42101 USA

**Keywords:** *Basilia*, *cox*1, *Cyclopodia*, Nycteribiidae, Bat flies, Madagascar, Comoros, Archipelago

## Abstract

**Background:**

We present information on Nycteribiidae flies parasitizing the bat families Pteropodidae, Miniopteridae and Vespertilionidae from the Malagasy Region, contributing insight into their diversity and host preference.

**Results:**

Our phylogenetic analysis identified nine clusters of nycteribiid bat flies on Madagascar and the neighbouring Comoros Archipelago. Bat flies sampled from frugivorous bats of the family Pteropodidae are monoxenous: *Eucampsipoda madagascariensis*, *E*. *theodori* and *Cyclopodia dubia* appear wholly restricted to *Rousettus madagascariensis*, *R*. *obliviosus* and *Eidolon dupreanum*, respectively. Two different host preference patterns occurred in nycteribiids infecting insectivorous bats. Flies parasitizing bats of the genera *Miniopterus* (Miniopteridae) and *Myotis* (Vespertilionidae), namely *Penicillidia leptothrinax*, *Penicillidia* sp. and *Nycteribia stylidiopsis*, are polyxenous and showed little host preference, while those parasitizing the genera *Pipistrellus* and *Scotophilus* (both Vespertilionidae) and referable to *Basilia* spp., are monoxenous. Lastly, the inferred Bayesian phylogeny revealed that the genus *Basilia*, as currently configured, is paraphyletic.

**Conclusion:**

This study provides new information on the differentiation of nycteribiid taxa, including undescribed species. Host preference is either strict as exemplified by flies parasitizing fruit bats, or more relaxed as found on some insectivorous bat species, possibly because of roost site sharing. Detailed taxonomic work is needed to address three undescribed nycteribiid taxa found on *Pipistrellus* and *Scotophilus*, tentatively allocated to the genus *Basilia*, but possibly warranting different generic allocation.

**Electronic supplementary material:**

The online version of this article (10.1186/s13071-017-2582-x) contains supplementary material, which is available to authorized users.

## Background

Information on bat diversity in the Malagasy Region (Madagascar and Comoros Archipelago) has increased considerably in recent decades with the description of several species new to science. Currently, 49 distinct bat species have been reported in this region, of which about 80% are endemic [1–4]. These investigations, which included new field collections of bats and their ectoparasites, have substantially clarified the taxonomy of the regional bat fauna and improved previously available information [5] on the diversity and ecology of bat parasites, including flies of the family Nycteribiidae [6, 7]. Nycteribiids are wingless pupiparous Diptera known to infest species of the bat suborders Yinpterochiroptera and Yangochiroptera [8]. Due to their obligatory parasitic lifestyle, nycteribiids live near their hosts, and different life history traits of bats presumably influence the ecology of these ectoparasites. Finally, the obligatory blood-feeding behaviour of nycteribiid flies may be important in structuring the diversity of associated bat microorganisms of possible medical importance [9, 10].

On Madagascar, previous studies of nycteribiid diversity [5, 11], along with molecular data [12], have provided an overview of host preference and aspects of their evolutionary history. The latter study revealed different patterns of host preference in five nycteribiid taxa, including *Eucampsipoda madagascarensis* and *E*. *theodori* known only from frugivorous bats, specifically *Rousettus madagascariensis* (Pteropodidae, Yinpterochiroptera) on Madagascar and *R*. *obliviosus* in the Comoros, respectively [12]. Further, this study suggested little host preference associated with *Nycteribia stylidiopsis*, *Penicillidia* sp. and *P*. *leptothrinax* occurring on insectivorous bats of the genus *Miniopterus* (Miniopteridae, Yangochiroptera). In addition to these taxa and based on specimens collected on Madagascar, Theodor [5] previously reported the presence of *Cyclopodia dubia* on *Eidolon dupreanum* (Pteropodidae), *Basilia* (*Paracyclopodia*) *madagascarensis* on *Scotophilus borbonicus* (probably syn. of *S*. *robustus*, see below) (Vespertilionidae, Yangochiroptera) and *Penicillidia decipiens* (host not identified).

We present additional molecular data regarding bat flies parasitizing *Eidolon dupreanum*, as well as insectivorous Malagasy bats of the family Vespertilionidae, specifically *Myotis goudoti*, *Scotophilus robustus*, *S*. *marovaza* and *Pipistrellus* cf. *hesperidus*. These data provide new insights into nycteribiid diversity and evolutionary history, and may be applicable to studies on the epidemiology of bat fly associated pathogens.

## Methods

### Sample collection and morphological characterization

Nycteribiid specimens used in the present study were obtained during field inventories of bats conducted for different research projects [12–16]. Bat ectoparasite collection methods, as well as their morphological identification, generally follow previous publications [5, 12]. For bat flies collected on *Eidolon dupreanum*, *Pipistrellus* cf. *hesperidus*, *Scotophilus marovaza* and *S*. *robustus*, morphological identification was undertaken using a published key [5] and confirmed by CWD. For each bat fly taxon, one specimen per host bat species was randomly selected for sequencing except flies parasitizing one individual of *S*. *marovaza* for which four flies were analyzed and included in a bacteriome study of nycteribiids from the Malagasy Region [10] (Additional file 1: Table S1).

### Molecular analyses

Whole specimens of bat flies or single intermediate legs were used for DNA extraction following described procedures [12]. Because previously published phylogenies of Malagasy bat flies that employed mitochondrial and nuclear markers were found to be congruent [12], we only amplified and sequenced a portion of the mitochondrial marker cytochrome *c* oxidase subunit 1 encoding gene (*cox*1). All new sequences generated in the present study (658 bp) are deposited in GenBank under accession numbers MF462026–MF462051 and were combined with sequences accessible through GenBank (Additional file 1: Table S1). Alignment was performed using MAFFT implemented in *Geneious Pro* version 6.1.4 (http://www.geneious.com [17]), and revealed no insertions or deletions. The selected best substitution model was based on Akaike Information Criterion as determined by jModelTest 2.1.3 [18, 19]. Subsequently, the Bayesian inference was conducted using MrBayes 3.1.2. [20]. This analysis consisted of two independent runs of four incremental Metropolis-Coupled Markov Chain Monte Carlo (MC^3^) iterations starting from a random tree. MC^3^ was calculated for 5,000,000 generations with trees and associated model parameters sampled every 500 generations. Further, pairwise genetic distances between sequences were calculated using the Kimura 2-parameter model [21] with bootstrapped replicates using MEGA 6.0 software [22].

### Host-parasite coevolution

We used ParaFit to test potential host-parasite coevolution between nycteribiid flies and bat host species; the null hypothesis was that evolution of hosts (bats) and parasites (nycteribiids) are independent [23]. As only a single sequence per lineage can be used as input in ParaFit, one consensus sequence was generated using the software *Geneious* for the well-supported clade of each parasite and available *cox*1 (658 bp) sequences. Because of major taxonomic revisions of Malagasy bats during the past decade, especially within the genus *Miniopterus*, we used only one recent sequence of cytochrome *b* (1047 bp) downloaded from GenBank for each bat host species. Phylogenies were generated using PhyML implemented in Seaview version 4 [24] and with 1000 replicates. The parafit test was performed using the *APE* package [25] under R version 3.0.0 [26]. Finally, a tanglegram allowing visualization of host-parasite associations was created with the software TreeMap 3b [27].

## Results

**Fig. 1:**
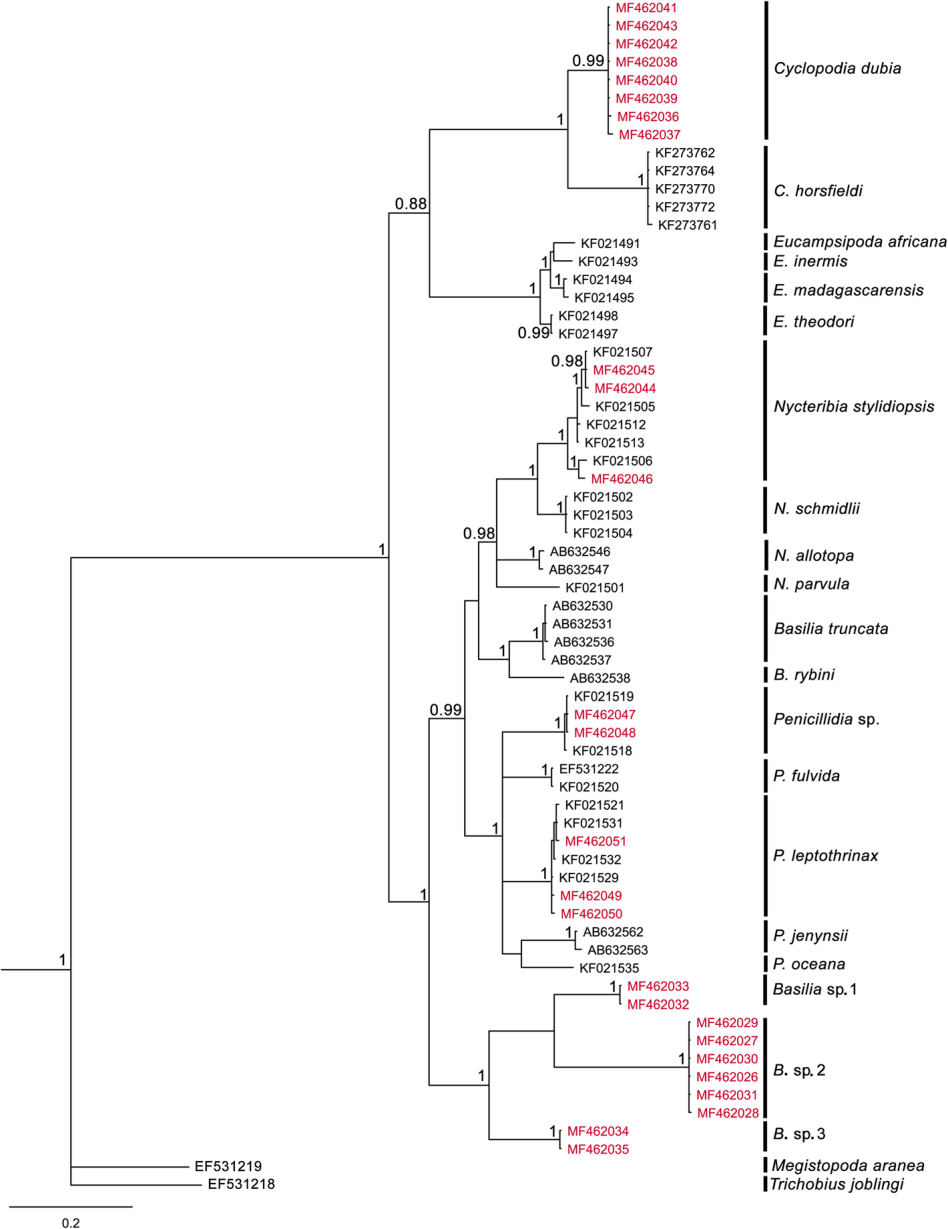
Phylogenetic relationships of nycteribiid flies parasitizing bats on Madagascar and the Comoros. The analysis was carried out using Bayesian inference under the GTR + I + G substitution model. Values in the nodes represent posterior probability, and only pp. > 0.80 are mentioned. Branchlets in red represent bat flies from the Malagasy Region. Generic abbreviations under the nycteribiid column include: *B*., *Basilia*; *C*., *Cyclopodia*; *E*., *Eucampsipoda*; *N*., *Nycteribia*; *P*., *Penicillidia*

Sixty-six sequences from nycteribiid bat fly sampled from different bat families, including 26 sequences produced in the context of this study, resulted in the differentiation of nine genetically distinct clades (Fig. 1), with differing patterns of host preference (Table 1).

**Table 1:** Nycteribiid species identified from different bat hosts from the Malagasy Region

Bats/Nycteribiids	*C. dubia*	*E. madagascarensis*	*E. theodori*	*Basilia* sp. 1	*Basilia* sp. 2	*Basilia* sp. 3	*N. stylidiopsis*	*P. leptothrinax*	*Penicillidia* sp.
*E. dupreanum*	×								
*R. madagascariensis*		×							
*R. obliviosus*			×						
*S. marovaza*					×				
*S. robustus*				×					
*P. hesperidus*						×			
*Myotis goudoti*							×		×
*M. aelleni*							×	×	
*M. griveaudi*							×	×	×
*M. gleni*							×	×	×
*M. mahafaliensis*								×	
*M. majori*							×	×	
*M. manavi*								×	×
*M. petersoni*							×	×	
*M. sororculus*							×	×	
Status	Monoxenous	Monoxenous	Monoxenous	Monoxenous	Monoxenous	Monoxenous	Polyxenous	Polyxenous	Polyxenous
Reference	This study	[12]	[12]	This study	This study	This study	[12]; this study	[12]	[12]; this study

### Phylogeny of Nycteribiidae bat flies

Bayesian inference based on mitochondrial sequences revealed that bat fly species parasitizing frugivorous (Pteropodidae) and insectivorous (Vespertilionidae, Miniopteridae) bats form two distinct well-supported monophyletic clades (posterior probability, pp = 1) (Fig. 1). Flies parasitizing frugivorous bats and belonging to the subfamily Cyclopodiinae [28] are divided into two well-supported clades (pp = 1) comprised of the genera *Cyclopodia* and *Eucampsipoda* found on *Eidolon dupreanum* and *Rousettus* spp., respectively. *Cyclopodia horsfieldi* parasitizing *Pteropus hypomelanus* from Malaysia falls within the first clade*.* Molecular data presented herein from flies collected on insectivorous bats from Madagascar and the Comoros Archipelago and belonging to the subfamily Nycteribiinae [29] also yielded two well-supported monophyletic clades composed of *Nycteribia* spp. and *Penicillidia* spp., parasitizing *Miniopterus* spp., as well as *Myotis goudoti*. Both *Nycteribia* spp. and *Penicillidia* spp. occurred on a variety of different host species and in certain cases, a single putative fly species was identified from several different bat species (Table 1). In addition, a separate and well-supported cluster of bat flies parasitizing Malagasy vespertilionids, specifically *Scotophilus robustus*, *S*. *marovaza*, and *Pipistrellus* cf. *hesperidus* (denoted as *Basilia* sp. 1–3 in Fig. 1) formed an independent, monophyletic group excluding *B*. *rybini* and *B*. *truncata* from Japan. In this phylogeny, the genus *Basilia* is paraphyletic and contains previously unrecognized diversity. Based on the Kimura 2-parameter model (K2P, Table 2), the average genetic distance between nycteribiid species from the Malagasy Region ranged from 3.0 to 20.4%. Bat flies infecting *S*. *robustus* and *S*. *marovaza* formed a monophyletic clade composed of two groups separated by a genetic distance of 12.1%, supporting the existence of two distinct species. Similarly, flies parasitizing *Pipistrellus* cf. *hesperidus* were notably divergent (12.1 to 13.5%) from those found on *Scotophilus* spp.

**Table 2:** Kimura 2-Parameter distances (in %, below diagonal) between groups as based on *cox*1 sequences (658 bp) and calculated using MEGA 6.0 [17] for nycteribiid species from the Malagasy Region; values above the diagonal represent the standard error (in %)

	Cdub	Bsp1	Bsp2	Bsp3	Nstyl	Psp	Plepto	Emad	Ethe
Cdub	–	1.6	1.9	1.5	1.6	1.7	1.7	1.5	1.6
Bsp1	17.3	–	1.5	1.4	1.5	1.5	1.6	1.6	1.6
Bsp2	20.4	12.1	–	1.5	1.4	1.9	1.9	1.7	1.8
Bsp3	15.3	11.5	13.5	–	1.5	1.6	1.5	1.6	1.6
Nstyl	15.8	14.3	16.7	12.6	–	1.6	1.3	1.6	1.6
Psp	17.6	14.9	17.9	14.0	12.0	–	1.1	1.7	1.7
Plepto	16.8	15.9	19.0	13.2	11.0	8.5	–	1.7	1.7
Emad	15.0	14.7	17.1	15.2	15.6	16.1	16.8	–	0.6
Ethe	14.8	15.5	17.8	14.6	15.6	15.8	15.9	3.0	–

*Abbreviations*: Cdub, *Cyclopodia dubia*; Bsp1, *Basilia* sp. 1; Bsp2, *Basilia* sp. 2; Bsp3, *Basilia* sp. 3; Nstyl, *Nycteribia stylidiopsis*; Psp, *Penicillidia* sp.; Plepto, *P. leptothrinax*; Emad, *Eucampsipoda madagascarensis*; Ethe, *E. theodori*

### Host-parasite coevolution

**Fig. 2:**
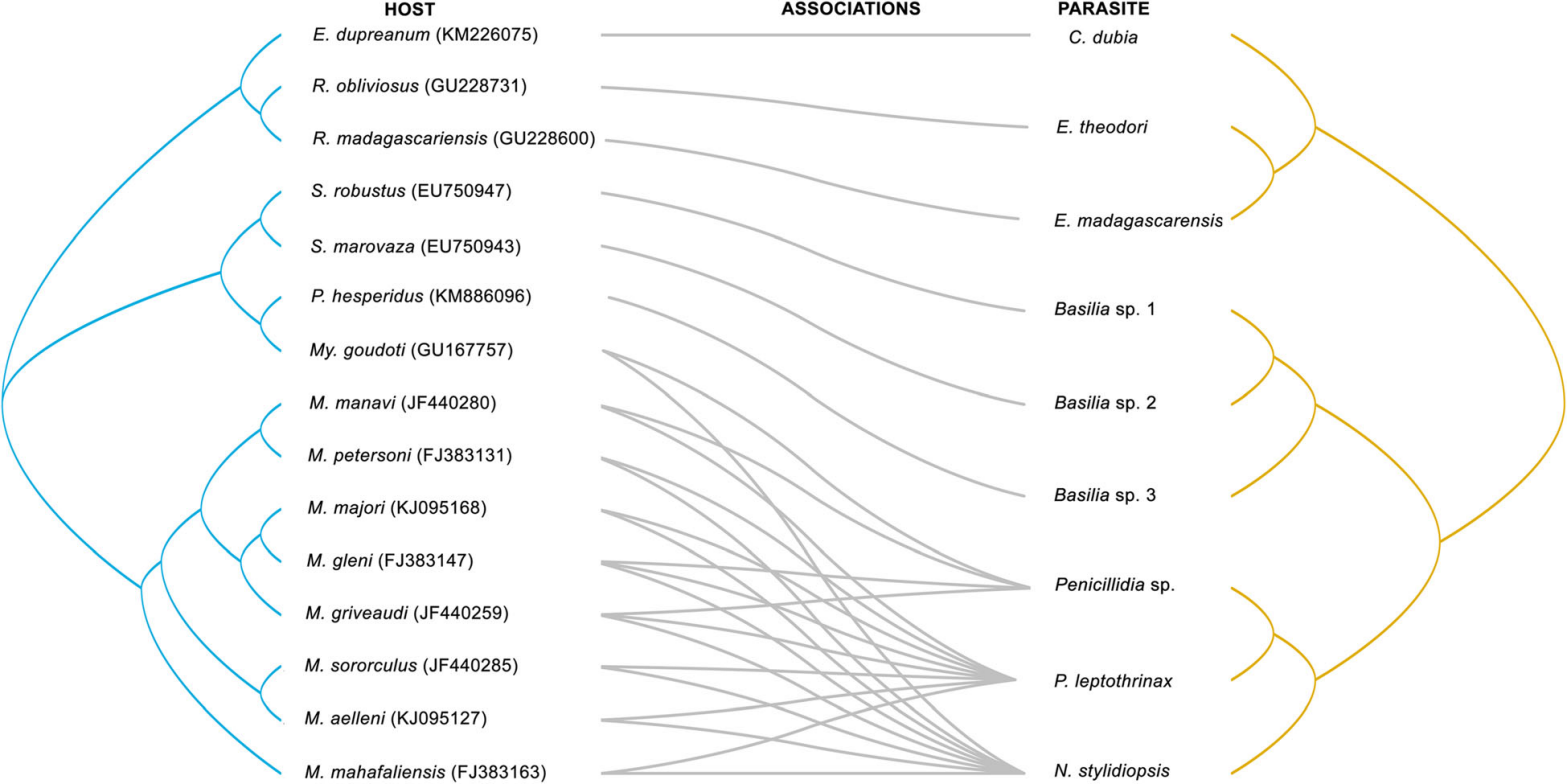
Host-parasite associations between bat host (*cytb*) and their nycteribiid flies (*cox*1). Generic abbreviations under the parasite column include: *B*., *Basilia*; *C*., *Cyclopodia*; *E*., *Eucampsipoda*; *N*., *Nycteribia*; *P*., *Penicillidia*. Abbreviations under the host column include: *E*., *Eidolon*; *M*., *Miniopterus*; *My.*, *Myotis*; *P*., *Pipistrellus*; *R*., *Rousettus*; *S*., *Scotophilus*

Overlaying the phylogeny of bat flies on that of their hosts (Fig. 2) provides a means to examine patterns of host-parasite associations and potential coevolutionary signal. Nycteribiid species parasitizing frugivorous bats appear to show one-to-one (monoxenous) associations indicative of strict host preference, while two distinct patterns emerge for nycteribiids parasitizing insectivorous bats: (i) flies infecting *Scotophilus* spp. and *Pipistrellus* cf. *hesperidus* are restricted to their respective host; and (ii) *Nycteribia stylidiopsis* and *Penicillidia* spp., parasitize *Myotis goudoti* and several *Miniopterus* spp.

ParaFit-based analysis of 15 bat hosts parasitized by nine nycteribiid taxa revealed a significant signal of phylogenetic congruence (putative host-parasite coevolution) between bats and their associated bat flies (ParaFitGlobal = 1.431, *P*-value = 0.001, 999 permutations). Out of the 26 host-parasite interactions, 24 showed significant associations, suggesting that the majority of the examined associations of nycteribiids and their bat hosts are not random. Two associations were found to be non-significant and only involved *Myotis goudoti*, parasitized by *Penicillidia* sp. and *Nycteribia stylidiopsis* (individual host-parasite link: *P*-value > 0.05) (Fig. 2).

## Discussion

The present study, based on molecular techniques, enriches information on diversity and host preference of Malagasy and Comorian bat flies of the family Nycteribiidae parasitizing bats of the families Pteropodidae, Miniopteridae, and Vespertilionidae. *Eucampsipoda madagascarensis* and *E*. *theodori* each parasitize a single species of pteropodid bat, *R. madagascariensis* from Madagascar and *R*. *obliviosus* from the Comoros, respectively. Similarly, *Cyclopodia dubia* was only associated with another Malagasy pteropodid species, *Eidolon dupreanum*. Based on available literature, these nycteribiid species appear to have strong host preference for single host species [5, 30]. Recent work on *Eucampsipoda madagascarensis* parasitizing *R. madagascariensis* in northern Madagascar found a skewed sex-ratio in favour of males and a reduced parasitism rate in the dry season (September) relative the wet season (January) [31]. Based on the available literature, not one of the nycteribiid species found on frugivorous bats was recorded from any animalivorous bat. In contrast, three taxa of nycteribiids, *Nycteribia stylidiopsis*, *Penicillidia* sp., and *P. leptothrinax*, were found to be polyxenous, parasitizing some bat host species, including non-congeners. However, their hosts are invariably insectivorous bat species, which often co-occupy the same day roost sites and are in close physical contact (i.e. roosting in syntopy). This phenomenon is best exemplified by the genus *Miniopterus*, which is comprised of at least 11 species endemic to the Malagasy Region, forming a monophyletic radiation [2] and, in different combinations, members of this genus often co-inhabit cave roosts with *Myotis goudoti*, a vespertilionid endemic to Madagascar [32]. The host-parasite analysis supports strong associations between *Miniopterus* spp. and *Nycteribia stylidiopsis*/*Penicillidia leptothrinax/Penicillidia* sp. but not between *Myotis goudoti* and *N. stylidiopsis*/*Penicillidia* sp. This pattern may be explained by host switching events of bat flies between *Miniopterus* spp*.* and *Myotis goudoti*. Similarly, such host switching events have been recently proposed to explain the current pathogenic bacteria diversity found in the same bat taxa [14]. Nonetheless, it is possible that some nycteribiid species may occasionally infest and take a blood meal from infected bats that are not their primary hosts, potentially explaining the occurrence of a given parasite in these nycteribiid flies [33]. Moreover, certain bat flies (e.g. the New World Streblidae) are known to display unexpectedly high levels of host preference [8, 34]. Hence, one explanation for the observation of the same parasite occurring on bats of different families centers on the concept of a “reproductive filter”, specifically, where newly emerged adult flies must locate a host with conspecific flies for mating and reproduction. Further, the presence of many potential hosts living in close contact within the same biotope will increase the probability of host switching [35]. In this case, the physical contact between *M*. *goudoti* and *Miniopterus* spp. in day roosts may weaken the effect of this reproductive filter and in turn, host preference. In any case, our results show that *Miniopterus* spp. and *Myotis goudoti* share the same bat fly species (*Penicillidia* sp. and *N. stylidiopsis*). For instance, *Penicillidia* sp. flies sampled on *M. goudoti* (MF462048), *M*. *manavi* (MF462047) and *M*. *griveaudi* (KF02519) showed the same identity based on *cox*1 sequences; similarly, *N*. *stylidiopsis* flies sampled on *M*. *goudoti* (MF462045), and *M*. *gleni* (KF021507) were identical based on this same marker. Interestingly, a recent genetic study on *Cyclopodia horsfieldi* from three species of *Pteropus* (*P*. *vampurus*, *P*. *hypomelanus* and *P*. *lylei*) in South East Asia (Cambodia, Vietnam and Malaysia) showed a low level of genetic structure within populations of this nycteribiid species. The lack of genetic structure across these host taxa and geography argues in favour of the role of *P*. *vampyrus*, with the broadest distribution, in the movement of bat flies between these *Pteropus* spp. [36]. In our case, such a mechanism could be proposed for certain bat taxa acting as dispersal bridges for bat flies and the likely candidates on Madagascar would be *Miniopterus* spp. and *Myotis goudoti*.

The lack of apparent nycteribiid host preference on *Miniopterus* spp. and *Myotis goudoti* may have consequences for the role of their fly parasites as potential reservoirs of different pathogens of medical or veterinary importance. It was recently shown that closely related bacteria belonging to the genus *Bartonella* were found in *N*. *stylidiopsis* and *P*. *leptothrinax* [10]. However, these types of switches might be more complex, as evidence of bacterial exchange between allopatric nycteribiid species has been reported, suggesting that different biological processes are involved in the structuring of microparasites relative to macroparasites. However, because the bats sampled were not screened for *Bartonella*, conclusions about these fly taxa as potential vectors of *Bartonella* are premature.

There is evidence that certain Malagasy *Miniopterus* spp. have evolved from sister species in geographical isolation, for example, *M. griffithsi* [2], while other species, such as *M*. *gleni*, together with *Myotis goudoti*, have broad geographical distributions [3] and show little phylogeographic structure [37, 38], presumably related to their dispersal patterns. Hence, it is possible that taxa such as *Myotis goudoti* and *Miniopterus gleni* act as bridge species allowing the dispersion of bat flies and associated microparasites between allopatric populations of *Miniopterus* spp.

The last cluster of nycteribiid flies infecting Malagasy bats belongs to the speciose and cosmopolitan genus *Basilia* (sensu *lato*). On Madagascar, the only currently recognized species is *B*. (*Paracyclopodia*) *madagascarensis*, collected from “*Scotophilus borbonicus*” on Nosy Be, a near-shore island in the northwest [5]. However, only two specimens tentatively identified as *S*. *borbonicus* are known from Madagascar [32] and based on the known distribution of members of this genus it is more likely that the host species was the Malagasy endemic *S*. *robustus*. Our analysis reveals paraphyly within *Basilia*. Malagasy bat fly species currently assigned to this genus show considerable genetic variation, ranging from 11.5 to 13.5% divergence (Table 2) and appear specific to their bat hosts (*S*. *robustus*, *S*. *marovaza*, and *Pipistrellus* cf. *hesperidus*, respectively). These results suggest that bat flies parasitizing Malagasy *Pipistrellus* and *Scotophilus* probably represent undescribed taxa that are potentially distinct from *Basilia*. Further, this genus is taxonomically complex and notably diverse [5, 11, 39, 40], and paraphyletic based on phylogenetic analyses [6]. Further systematic work is needed to resolve the generic and species classification of the divergent Malagasy taxa currently assigned to *Basilia*.

## Conclusions

The present study complements existing information on nycteribiid diversity and their host associations from the Malagasy Region and highlights the importance of bat ecology, particularly roosting habits, on the host preference of their parasites. These results underline the importance of multidisciplinary investigations involving mammalogists, entomologists, and microbiologists for a comprehensive understanding of the distribution, transmission, and potential spillover of bat-borne parasites and pathogens. Additionally, future systematic work must be conducted to identify nycteribiid flies in the other Malagasy Region bat families (e.g. Hipposideridae, Rhinonycteridae, Myzopodidae and Emballonuridae) that were not sampled in the present study. The availability of a detailed database on the bat flies in the region will help provide more comprehensive information on their distribution and patterns of host preference. Further, additional efforts may help to determine rates of parasitism, and how the dynamics of parasitism vary across the season in different populations of bat flies.

## Additional file

Additional file 1: Table S1. Nycteribiidae specimens and sequences used in this study, including isolates, GenBank accession numbers, host, and origin. Molecular data produced in the frame of the present work are marked with an asterisk (*). *Abbreviations*: FMNH, Field Museum of Natural History; KU, University of Kansas Natural History Museum; UADBA, Université d’Antananarivo, Département de Biologie Animale; NA, not available. (DOC 108 kb)

## References

[1] Goodman SM, Weyeneth N, Ibrahim Y, Saïd I, Ruedi M (2010). A review of the bat fauna of the Comoro archipelago. Acta Chiropt.

[2] Christidis L, Goodman SM, Naughton K, Appleton B (2014). Insights into the evolution of a cryptic radiation of bats: dispersal and ecological radiation of Malagasy *Miniopterus* (Chiroptera: Miniopteridae). PLoS One.

[3] Goodman SM, Ramasindrazana B, Goodman SM, Raherilalao MJ (2013). Bats or the order Chiroptera. Atlas of selected land vertebrates of Madagascar.

[4] Goodman SM, Rakotondramanana CF, Ramasindrazana B, Kearney T, Monadjem A, Schoeman MC (2015). An integrative approach to characterize Malagasy bats of the subfamily Vespertilioninae gray, 1821, with the description of a new species of *Hypsugo*. Zool J Linnean Soc.

[5] Theodor O (1957). The Nycteribiidae of Ethiopian region and Madagascar. Parasitology.

[6] Dittmar K, Porter ML, Murray SW, Whiting MF (2006). Molecular phylogenetic analysis of nycteribiid and streblid bat flies (Diptera: Brachycera, Calyptratae): implications for host associations and phylogeographic origins. Mol Phyl Evol.

[7] Dittmar K, Morse SF, Dick CW, Patterson BD, Morand S, Krasnov BR, Littlewood TDJ (2015). Bat fly evolution from the Eocene to the present (Hippoboscoidea, Streblidae and Nycteribiidae). Parasite diversity and diversification: evolutionary ecology meets phylogenetics.

[8] Dick CW, Patterson BD, Morand S, Krasnov BR, Poulin R (2006). Bat flies - obligate ectoparasites of bats. Micromammals and macroparasites: from evolutionary ecology to management.

[9] Morse SF, Olival KJ, Kosoy M, Billeter S, Patterson BD, Dick CW, Dittmar K (2012). Global distribution and genetic diversity of *Bartonella* in bat flies (Hippoboscoidea, Streblidae, Nycteribiidae). Infect Genet Evol.

[10] Wilkinson DA, Duron O, Cordonin C, Gomard Y, Ramasindrazana B, Mavingui P (2016). The bacteriome of bat flies (Nycteribiidae) from the Malagasy region: a community shaped by host ecology, bacterial transmission mode, and host-vector specificity. Appl Environ Microbiol.

[11] Theodor O (1968). New species and new records of Nycteribiidae from Ethiopian. Oriental and Pacific regions Parasitology.

[12] Tortosa P, Dsouli N, Gomard Y, Ramasindrazana B, Dick CW, Goodman SM (2013). Evolutionary history of Indian Ocean nycteribiid bat flies mirroring the ecology of their hosts. PLoS One.

[13] Wilkinson DA, Mélade J, Dietrich M, Ramasindrazana B, Soarimalala V, Lagadec E (2014). Highly diverse Morbillivirus-related paramyxoviruses in the wild fauna of southwestern Indian Ocean islands: evidence of exchange between introduced and endemic mammals. J Virol.

[14] Gomard Y, Dietrich M, Wieseke N, Ramasindrazana B, Lagadec E, Goodman SM (2016). Malagasy bats shelter a considerable genetic diversity of pathogenic *Leptospira* suggesting notable host-specificity patterns. FEMS Microbiol Ecol.

[15] Ramasindrazana B, Dellagi K, Lagadec E, Randrianarivelojosia M, Goodman SM, Tortosa P (2016). Diversity, host specialization, and geographic structure of filarial nematodes infecting Malagasy bats. PLoS One.

[16] Mélade J, Wieseke N, Ramasindrazana B, Flores O, Lagadec E, Gomard Y (2016). An eco-epidemiological study of morbilli-related paramyxovirus infection in Madagascar bats reveals host-switching as the dominant macro-evolutionary mechanism. Sci Rep.

[17] Kearse M, Moir R, Wilson A, Stones-Havas S, Cheung M, Sturrock S (2012). Geneious basic: an integrated and extendable desktop software platform for the organization and analysis of sequence data. Bioinformatics.

[18] Posada D (2008). jModelTest: Phylogenetic model averaging. Mol Biol Evol.

[19] Darriba D, Taboada GL, Doallo R, Posada D (2012). jModelTest 2: more models, new heuristics and parallel computing. Nat Methods.

[20] Ronquist F, Teslenko M, Van Der Mark P, Ayres DL, Darling A, Höhna S (2012). MrBayes 3.2: efficient Bayesian phylogenetic inference and model choice across a large model space. Syst Biol.

[21] Kimura M (1980). A simple method for estimating evolutionary rates of base substitutions through comparative studies of nucleotide sequences. J Mol Evol.

[22] Tamura K, Stecher G, Peterson D, Filipski A, Kumar S (2013). MEGA6: Molecular Evolutionary Genetics Analysis version 6.0.. Mol Biol Evol.

[23] Legendre P, Desdevises Y, Bazin E (2002). A statistical test for host-parasite coevolution. Syst Biol.

[24] Gouy M, Guindon S, Gascuel O (2010). SeaView version 4: a multiplatform graphical user interface for sequence alignment and phylogenetic tree building. Mol Biol Evol.

[25] Paradis E, Claude J, Strimmer K (2004). Analyses of phylogenetics and evolution in R language. Bioinformatics.

[26] R: A language and environment for statistical computing. Vienna: R Foundation for Statistical Computing; 2013. Available at http://www.R-project.org.

[27] Charleston MA. TreeMap 3b. 2011. Available at http://sites.google.com/site/cophylogeny.

[28] Maa TC (1965). An interim world list of batflies. J Med Entomol.

[29] Westwood JO (1835). On Nycteribia, a genus of wingless insects. Trans Zool Soc London.

[30] Wenzel RL, Tipton VJ, Kiewlicz A, Wenzel RL, Tipton VJ (1966). The streblid batflies of Panama (Diptera Calypterae: Streblidae). Ectoparasites of Panama.

[31] Rajemison FI, Noroalintseheno LOS, Goodman SM (2017). Bat flies (Diptera: Nycteribiidae, Streblidae) parasitising *Rousettus madagascariensis* (Chiroptera: Pteropodidae) in the Parc national d'Ankarana: species diversity, rates of parasitism and sex ratios. Afr Entomol.

[32] Goodman SM (2011). Les chauves-souris de Madagascar.

[33] Obame-Nkoghe J, Rahola N, Bourgarel M, Yangari P, Prugnolle F, Maganga GD, et al. Bat flies (Diptera: Nycteribiidae and Streblidae) infesting cave-dwelling bats in Gabon: diversity, dynamics and potential role in *Polychromophilus melanipherus* transmission. Parasit Vectors. 2016;9:333. 10.1186/s13071-016-1625-z PMC4902993 27286888

[34] Dick CW (2007). High host specificity of obligate ectoparasites. Ecol Entomol.

[35] Dick CW, Patterson BD (2007). Against all odds: explaining high host specificity in dispersal-prone parasites. Int J Parasitol.

[36] Olival KJ, Dick CW, Simmons NB, Morales JC, Melnick DJ, Dittmar K (2013). Lack of population genetic structure and host specificity in the bat fly, *Cyclopodia horsfieldi*, across species of *Pteropus* bats in Southeast Asia. Parasit Vectors.

[37] Weyeneth N, Goodman SM, Ruedi M (2011). Do diversification models of Madagascar’s biota explain the population structure of the endemic bat *Myotis goudoti* (Chiroptera: Vespertilionidae)?. J Biogeogr.

[38] Goodman SM, Maminirina CP, Bradman HM, Christidis L, Appleton BR (2010). Patterns of morphological and genetic variation in the endemic Malagasy bat *Miniopterus gleni* (Chiroptera: Miniopteridae), with the description of a new species, *M. griffithsi*. J Zool Syst Evol Res.

[39] Graciolli G, Dick CW (1972). A new species of *Basilia* Miranda-Ribeiro (Diptera: Nycteribiidae) from Honduras, parasite of *Bauerus dubiaquercus* (van Gelder) (Chiroptera: Vespertilionidae: Antrozoinae). Zootaxa.

[40] Maa T (1968). New *Basilia* species from Thailand, Mexico and Brazil (Diptera: Nycteribiidae). Pacific Insects.

[41] Sikes RS, Gannon WL (2011). The animal care and use Committee of the American Society of Mammalogists: guidelines of the American Society of Mammalogists for the use of wild mammals in research. J Mammal.

