# Supplementary material for: Hidden diversity of Nycteribiidae (Diptera) bat flies from the Malagasy region and insights on host-parasite interactions
Source: Parasit Vectors. 2017 Dec 29;10:630. doi: 10.1186/s13071-017-2582-x (PMC5747079; doi:10.1186/s13071-017-2582-x)
Supplement: Additional file 1: Table S1. — Nycteribiidae specimens and sequences used in this study, including isolates, GenBank accession numbers, host, and origin. Molecular data produced in the frame of the present work are marked with an asterisk (*). Abbreviations: FMNH, Field Museum of Natural History; KU, University of Kansas Natural History Museum; UADBA, Université d’Antananarivo, Département de Biologie Animale; NA, not available. (DOC 108 kb) [file 13071_2017_2582_MOESM1_ESM.doc]

**Additional file 1: Table S1** Nycteribiidae specimens and sequences used in this study, including isolates, GenBank accession numbers, host, and origin. Molecular data produced in the frame of the present work are marked with an asterisk (*). *Abbreviations*: FMNH, Field Museum of Natural History; KU, University of Kansas Natural History Museum; UADBA, Université d’Antananarivo, Département de Biologie Animale; NA, not available.

| **Nycteribiidae** |  | **Accession number** | **Host** | | |
| --- | --- | --- | --- | --- | --- |
| **Species** | **Isolate** | **COI** | **Species** | **Museum voucher** | **Origin** |
| *Basilia* sp. 2* | SC1 | MF462026 | *Scotophilus marovaza* | FMNH 221393 | Madagascar |
| *Basilia* sp. 2* | SC2 | MF462027 | *Scotophilus marovaza* | FMNH 221393 | Madagascar |
| *Basilia* sp. 2* | SC3 | MF462028 | *Scotophilus marovaza* | FMNH 221393 | Madagascar |
| *Basilia* sp. 2* | SC4 | MF462029 | *Scotophilus marovaza* | FMNH 221393 | Madagascar |
| *Basilia* sp. 2* | N18 | MF462030 | *Scotophilus marovaza* | FMNH 194546 | Madagascar |
| *Basilia* sp. 2* | N19 | MF462031 | *Scotophilus marovaza* | FMNH 194549 | Madagascar |
| *Basilia* sp. 1* | N11 | MF462032 | *Scotophilus robustus* | FMNH 188063 | Madagascar |
| *Basilia* sp. 1* | 1B | MF462033 | *Scotophilus robustus* | FMNH 209163 | Madagascar |
| *Basilia* sp. 3* | Bas3a | MF462034 | *Pipistrellus* cf. *hesperidus* | UADBA 32935 | Madagascar |
| *Basilia* sp. 3* | Bas3b | MF462035 | *Pipistrellus* cf. *hesperidus* | UADBA 32936 | Madagascar |
| *Basilia truncata* | BaTr7 | AB632536 | *Myotis mystacinus* | NA | Japan |
| *Basilia truncata* | BaTr1 | AB632530 | *Plecotus auritus* | NA | Japan |
| *Basilia truncata* | BaTr2 | AB632531 | *Barbastella leucomelas* | NA | Japan |
| *Basilia truncata* | BaTr8 | AB632537 | *Myotis ikkinikovi* | NA | Japan |
| *Basilia rybini* | BaRy1 | AB632538 | *Myotis daubentonii* | NA | Japan |
| *Cyclopodia dubia** | 1Cdu | MF462036 | *Eidolon dupreanum* | FMNH 221295 | Madagascar |
| *Cyclopodia dubia** | 2Cdu | MF462037 | *Eidolon dupreanum* | FMNH 221296 | Madagascar |
| *Cyclopodia dubia** | 3Cdu | MF462038 | *Eidolon dupreanum* | FMNH 221297 | Madagascar |
| *Cyclopodia dubia** | 4Cdu | MF462039 | *Eidolon dupreanum* | FMNH 221298 | Madagascar |
| *Cyclopodia dubia** | 5Cdu | MF462040 | *Eidolon dupreanum* | UADBA 32975 | Madagascar |
| *Cyclopodia dubia** | 6Cdu | MF462041 | *Eidolon dupreanum* | UADBA 32976 | Madagascar |
| *Cyclopodia dubia** | 7Cdu | MF462042 | *Eidolon dupreanum* | UADBA 32977 | Madagascar |
| *Cyclopodia dubia** | 9Cdu | MF462043 | *Eidolon dupreanum* | UADBA 32979 | Madagascar |
| *Cyclopodia horsfieldi* |  | KF273761 | *Pteropus hypomelanus* |  | Malaysia |
| *Cyclopodia horsfieldi* |  | KF273762 | *Pteropus hypomelanus* |  | Malaysia |
| *Cyclopodia horsfieldi* |  | KF273764 | *Pteropus hypomelanus* |  | Malaysia |
| *Cyclopodia horsfieldi* |  | KF273770 | *Pteropus hypomelanus* |  | Malaysia |
| *Cyclopodia horsfieldi* |  | KF273772 | *Pteropus hypomelanus* |  | Malaysia |
| *Eucampsipoda africana* | N4 | KF021491 | *Rousettus aegyptiacus* | FMNH 215397 | Kenya |
| *Eucampsipoda inermis* | N8 | KF021493 | *Rousettus amplexicaudatus* | KU 165172 | Philippines |
| *Eucampsipoda madagascarensis* | J43 | KF021494 | *Rousettus madagascariensis* | UADBA 43757 | Madagascar |
| *Eucampsipoda madagascarensis* | J48 | KF021495 | *Rousettus madagascariensis* | FMNH 209105 | Madagascar |
| *Eucampsipoda theodori* | 29F | KF021497 | *Rousettus obliviosus* | FMNH 220041 | Comoros |
| *Eucampsipoda theodori* | 30DM | KF021498 | *Rousettus obliviosus* | FMNH 220042 | Comoros |
| *Nycteribia allotopa* |  | AB632546 | *Miniopterus fuliginosus* | NA | Japan |
| *Nycteribia allotopa* |  | AB632547 | *Miniopterus fuliginosus* | NA | Japan |
| *Nycteribia parvula* | N16 | KF021501 | *Miniopterus schreibersii* | KU 165042 | Philippines |
| *Nycteribia schmidlii* | N21 | KF021502 | *Miniopterus africanus* | FMNH 215719 | Kenya |
| *Nycteribia schmidlii* | N2 | KF021503 | *Miniopterus inflatus* | FMNH 215674 | Kenya |
| *Nycteribia schmidlii* | N3 | KF021504 | *Miniopterus africanus* | FMNH 215697 | Kenya |
| *Nycteribia stylidiopsis* | 33A | KF021505 | *Miniopterus griveaudi* | FMNH 220061 | Comoros |
| *Nycteribia stylidiopsis* | 42A | KF021506 | *Miniopterus petersoni* | FMNH 209186 | Madagascar |
| *Nycteribia stylidiopsis* | Nstyl1 | MF462044 | *Miniopterus aelleni* | FMNH 184065 | Madagascar |
| *Nycteribia stylidiopsis* | GR12 | KF021507 | *Miniopterus gleni* | FMNH 221333 | Madagascar |
| *Nycteribia stylidiopsis* | J32 | KF021512 | *Miniopterus sororculus* | UADBA 43264 | Madagascar |
| *Nycteribia stylidiopsis* | J33 | KF021513 | *Miniopterus majori* | FMNH 209177 | Madagascar |
| *Nycteribia stylidiopsis** | 10B | MF462045 | *Myotis goudoti* | UADBA 33000 | Madagascar |
| *Nycteribia stylidiopsis** | Nstyl2 | MF462046 | *Myotis goudoti* | UADBA 50345 | Madagascar |
| *Penicillidia* sp. | J68 | KF021518 | *Miniopterus gleni* | FMNH 218031 | Madagascar |
| *Penicillidia* sp. | GR21 | KF021519 | *Miniopterus griveaudi* | FMNH 221337 | Madagascar |
| *Penicillidia* sp.* | 9B | MF462047 | *Miniopterus* cf. *manavi* | FMNH 221423 | Madagascar |
| *Penicillidia* sp.* | Psp | MF462048 | *Myotis goudoti* | FMNH 221420 | Madagascar |
| *Penicillidia fulvida* | N20 | KF021520 | *Miniopterus africanus* | FMNH 215688 | Kenya |
| *Penicillidia fulvida* |  | EF531222 | Unidentified bat | NA | NA |
| *Penicillidia jenynsii* |  | AB632562 | *Miniopterus fuliginosus* | NA | Japan |
| *Penicillidia jenynsii* |  | AB632563 | *Miniopterus fuliginosus* | NA | Japan |
| *Penicillidia leptothrinax* | GR7 | KF021521 | *Miniopterus griveaudi* | FMNH 221405 | Madagascar |
| *Penicillidia leptothrinax* | J34 | KF021529 | *Miniopterus petersoni* | FMNH 209186 | Madagascar |
| *Penicillidia leptothrinax** | 17B | MF462049 | *Miniopterus* cf. *manavi* | FMNH 221423 | Madagascar |
| *Penicillidia leptothrinax** | 11C | MF462050 | *Miniopterus aelleni* | FMNH 221440 | Madagascar |
| *Penicillidia leptothrinax** | J27 | MF462051 | *Miniopterus manavi* | FMNH 209179 | Madagascar |
| *Penicillidia leptothrinax* |  | KF021532 | *Miniopterus mahafaliensis* | UADBA 50161 | Madagascar |
| *Penicillidia leptothrinax* |  | KF021531 | *Miniopterus gleni* | UADBA 50174 | Madagascar |
| *Penicillidia oceana* | N17 | KF021535 | *Miniopterus schreibersii* | KU 165154 | Philippines |
| *Megistopoda aranea* |  | EF531219 | Bat | NA | NA |
| *Trichobius joblingi* |  | EF531218 | Bat | NA | NA |
